# Additively Manufactured Rotating Disk Electrodes and Experimental Setup

**DOI:** 10.1021/acs.analchem.2c02884

**Published:** 2022-09-21

**Authors:** Matthew J. Whittingham, Robert D. Crapnell, Craig E. Banks

**Affiliations:** Faculty of Science and Engineering, Manchester Metropolitan University, Chester Street, Manchester M1 5GD, United Kingdom

## Abstract

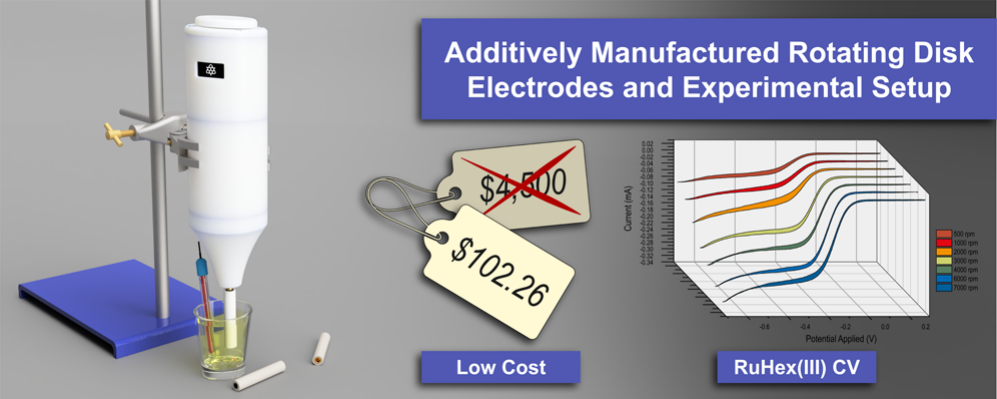

This manuscript details the first report of a complete additively manufactured rotating disk electrode setup, highlighting how high-performing equipment can be designed and produced rapidly using additive manufacturing without compromising on performance. The additively manufactured rotating disk electrode system was printed using a predominantly acrylonitrile butadiene styrene (ABS) based filament and used widely available, low-cost electronics, and simplified machined parts to create. The additively manufactured rotating disk electrode system costs less than 2% of a comparable commercial solution (£84.47 ($102.26) total). The rotating disk electrode is also additively manufactured using a carbon black/polylactic acid (CB/PLA) equivalent, developing a completely additively manufactured rotating disk electrode system. The electrochemical characterization of the additively manufactured rotating disk electrode setup was performed using hexaamineruthenium(III) chloride and compared favorably with a commercial glassy carbon electrode. Finally, this work shows how the additively manufactured rotating disk electrode experimental system and additive manufactured electrodes can be utilized for the electroanalytical determination of levodopa, a drug used in the treatment of Parkinson’s disease, producing a limit of detection of 0.23 ± 0.03 μM. This work represents a step-change in how additive manufacturing can be used in research, allowing the production of high-end equipment for hugely reduced costs, without compromising on performance. Utilizing additive manufacturing in this way could greatly enhance the research possibilities for less well-funded research groups.

## Introduction

The rotating disk electrode (RDE) is a classical hydrodynamic electrochemical technique based on controlled convective mass transport, in which the working electrode is rotated, creating a constant laminar flow of analyte to the electrode’s active surface.^1,2^ The rates of mass transport not only are controlled but can be readily altered giving a wide range of reaction time scales and can be explored in which kinetic and mechanism information may be probed.^1−3^ Typically, RDE are comprised of a disk of active material - Pt, Ni, Cu, Au, Fe, Si, CdS, GaAs, glassy carbon or graphite—set into an insulating (PTFE) surround. Other materials have been utilised, such as boron-doped diamond.^4^ These experimental systems can be prohibitively expensive (especially for less established research groups) costing thousands of pounds commercially, albeit for excellent quality. Production of this type of kit in-house would previously have required access to heavy engineering equipment; however additive manufacturing (AM) has removed the requirement for this and made the production of bespoke equipment much more accessible.

Additive manufacturing (AM) is quickly becoming a staple of modern life. While initially created for rapid-prototyping, AM has quickly broken through to multiple industries and increasingly households. It allows users to quickly move from a computer aided design (CAD) model to a physical object, in a “bottom-up” process in which the object is created by the addition (deposition) of layers of material. Many types of AM exist, from the common fused filament fabrication (FFF/FDM) and digital light processing (DLP) machines to industrial laminated object manufacturing (LOM), stereolithography (SLA), and selective laser sintering (SLS) machines. Through the use of an additive process (rather than traditional subtractive processes) waste material is almost eliminated; complex geometries can be produced, and no additional tooling is required.^5^ The cost of adoption of AM has historically been high; however with the surge in popularity of 3D-printing (3DP), industry growth, and vast competition in the market; the entry cost for AM has plummeted. Capable FFF machines are now readily available for sub £400, allowing individual researchers and small research groups to invest in AM equipment that was previously unobtainable.

AM (particularly FFF/FDM) has been applied to electrochemistry,^6^ with the research predominantly focused on the development and use of conductive materials for applications in the fields of energy storage^7−9^ and sensing.^10−12^ This area of research started with the production of “lollipop” electrodes from commercially available filament and has progressed into full electrochemical cells with embedded electrodes^13^ and the development of bespoke filaments.^14,15^ Additionally, there is a large amount of research being done to improve the sustainability of AM, through the recycling of used prints or reuse of other polymers into FFF printable filaments.^16,17^ The entry cost barrier for electrochemical equipment can be vastly reduced through the use of localized additive manufacture, as shown by previous research into the production of cells, electrodes, and accessories for the electrochemical lab,^18^ which showed various low-cost AM equipment, such as a screen-printed electrode (SPE) connector, in which it was notably cheaper to buy a 3D printer and produce the SPE holder for less cost than a single commercial SPE holder.

In this paper, we pose the question: Why cannot this approach be applied to lab analytical equipment itself? The RDE is a functionally simple piece of equipment. A (usually DC) motor drives a shaft, on which an electrode is attached, and the shaft is electrically connected to the electrode surface. This shaft is electrically contacted (usually through mercury or carbon–silver brushes) to the output of the equipment, which is in turn connected to a potentiostat. There has been sparse research into producing RDEs in situ, but this research wholly focuses on the electrodes, mounting/sealing solutions,^19^ or method of rotation.^2^ The cost of these solutions is almost never considered, making them potentially inaccessible to less well-funded researchers. AM removes this cost barrier in the aforementioned research, and so logically, it follows that AM could remove the cost barrier for RDE research.

## Methods

### Materials

All chemicals used were of analytical grade and used as received without any further purification. All solutions were prepared with deionized water of resistivity not less than 18.2 MΩ cm. Hexaamineruthenium(III) chloride (RuHex), 3,4-dihydroxy-l-phenylalanine (L-DOPA), and sodium hydroxide were purchased from Merck (Gillingham, U.K.). Potassium chloride was purchased from Fisher Scientific (Loughborough, U.K.).

The commercial conductive PLA/carbon black (PLA/CB) filament was purchased from Farnell (Leeds, U.K.). Note that we have utilized this filament in a different system and more details of its physiochemical characterized can be found in ref (18). Raise3D premium PLA filament and Raise3D premium ABS filament were purchased from Create Education (Chorley, U.K.). Flexible motor cage was printed from RS PRO 1.75 mm natural FLEX 45 purchased from RS (Corby, U.K.). 2 mm nitrile rubber O-ring stock, 5 mm i.d., 11 mm o.d. flanged ball bearings, 5 mm brass rod, M2.5 × 12 screws, M3 × 3 grub screws, M3 × 10 screws, banana jack socket for potentiostat connection, Arduino Nano development board, 2.1 mm barrel jack, and 200k trim potentiometer were purchased from RS (Corby, U.K.). The 12 V 10k rpm DC motor with encoder, carbon brushes, L298N module H bridge motor driver board, and SSD1306 128 × 64 OLED display were purchased from Amazon.

### Coding of Rotating Disk Electrode (RDE)

**Figure 1:**
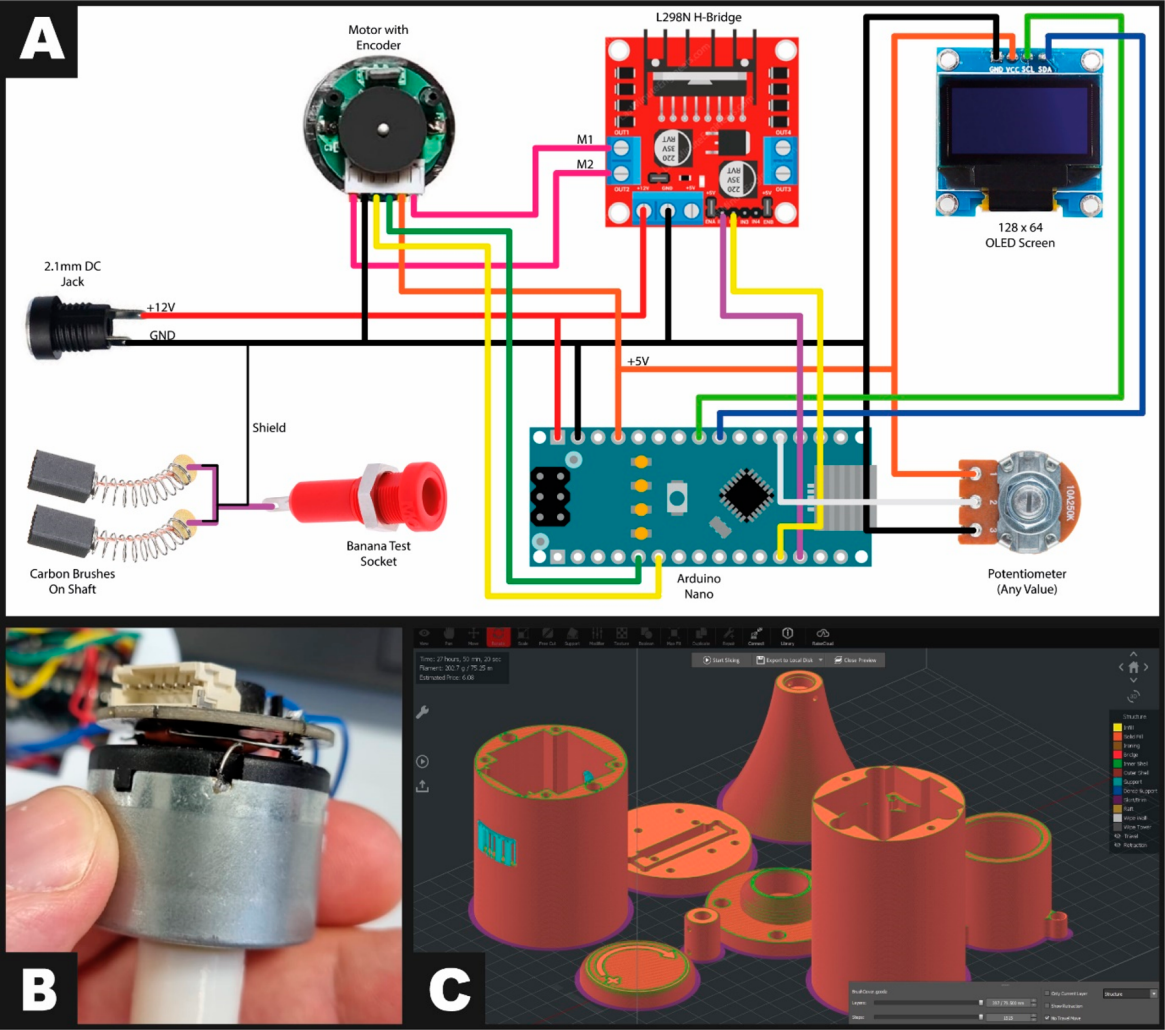
(A) Electronic construction of additively manufactured rotation disk electrode setup. (B) Ceramic capacitors connected between motor terminals and from each terminal to the motor casing/ground. (C) Screen capture of the IdeaMaker generated .GCODE file of the additively manufactured rotation disk electrode setup.

The use of an Arduino Nano as the RDE’s controller allows programming through Arduino IDE, a user-friendly and basic development environment. The system was wired as shown in Figure 1A. Note that the carbon brushes responsible for collecting the signal from the electrode are a separate circuit to the control system (with a shielded cable connected at one end to the control system’s ground) to prevent induced electrical noise from the motor reaching the output. Not pictured in Figure 1A are three 100 nF ceramic capacitors, soldered across the motor’s terminals and from each terminal to the motor’s metal casing, again to reduce electrical noise in the system, shown in Figure 1B. The code can be found in the Supporting Information and uses a logical progression to control the system:

- Libraries for AVR interrupts, I2C, SSD1306 screens are called.
- OLED screen is initialized.
- Variables and integers are defined.
- ISRs are created.
- Arduino (motor driver connections are defined as PWM pins) is for speed control. This is done using high level code to reduce overhead.
- PWM frequency is set to 20 kHz to reduce audible motor noise.
- External interrupt pins (from the motor encoder) are created, again using high level code.
- Serial connection is started, for USB debugging in case of errors.
- Screen initialization is checked.
- Boot screen is displayed.
- Main loop is started:

- Simple function to calculate the rpm of the motor and shaft.
- Show this calculated value on the OLED screen.
- Measure the difference between 5 and 0 V created by the potentiometer wired as a potential divider.
- Map this measured difference range (min/max on the knob) to a PWM value between ∼64 and 255, with 64 being the tested stall limit of the motor.
- Set L298N enable pin to low-set motor direction.
- Write above PWM value to PWM input of motor controller in an uninterruptable way.

- While simultaneously:

- Creating an easy-to-read display “3D-PRINTED RDE SYSTEM”, “XXXX” (calculated rpm value), “ RPM”.
- Update this display constantly with new rpm value.

### Additive Manufacturing (3D Printing)

ABS (acrylonitrile butadiene styrene) was chosen as the main RDE housing material, due to its high strength and temperature resistance, ease of printing, and ease of postprocessing, as well as its ability to be sterilized using EtO, formalin, or radiation. Note that any rigid filament would be suitable for the housing of the RDE, though postprocessing would vary between materials, as would chemical resistance. The parts were exported from Fusion360 as 0.3MF files, imported to Raise3D ideaMaker, sliced and then exported as .GCODE to a USB drive, Figure 1C. This .GCODE was then printed using a Raise3D E2 3d-printer, using a layer height of 0.2 mm, 4 walls, and 20% infill. The motor housing and shaft coupler were the only parts to differ from this, with the motor housing being printed from RS Pro Flex 45 with a 10% gyroid infill to promote flexibility and the shaft coupler being printed in solid ABS for strength. The modular housing was assembled and then smoothed with emery paper to produce a smooth surface finish without layer lines. The files needed to produce these prints can be found through a link in the Supporting Information.

### Electrochemical System

All electrochemical experiments were performed using a three-electrode system with either a glassy carbon electrode (GCE) or additively manufactured electrode (AME) as the working electrode, a nichrome wire as the counter electrode, and an Ag|AgCl electrode as the reference electrode, all controlled by an Autolab PGSTAT100N (Utrecht, The Netherlands) potentiostat. Solutions of RuHex were degassed thoroughly before experiments for at least 15 min with N_2_ gas.

## Results and Discussion

### Design, Production, and Cost of the Additive Manufacturing Rotating Disk Electrode (AMRDE)

**Figure 2:**
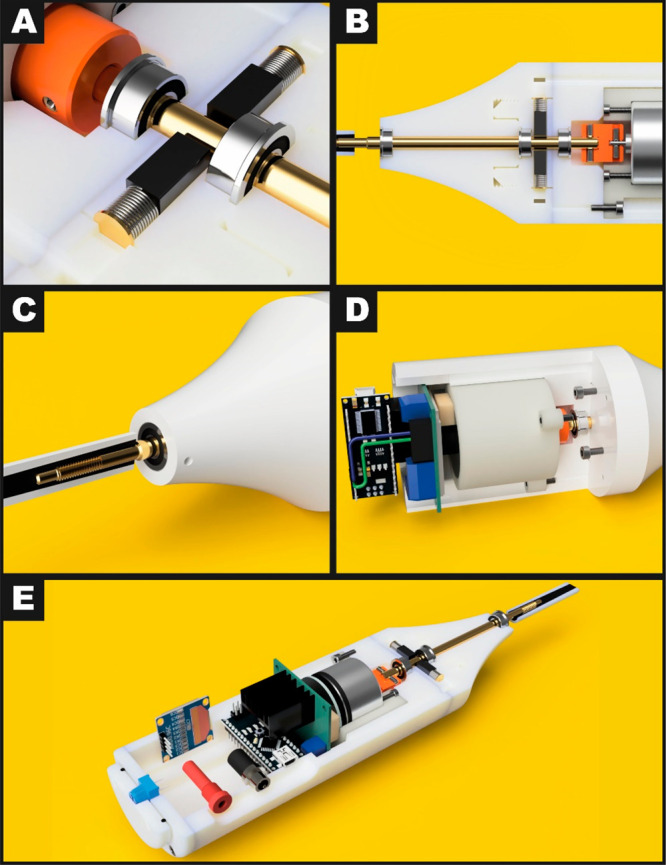
Design of additively manufactured rotating disk electrode setup. (A) Cross-sectional CAD view of carbon brush configuration and shaft coupler. (B) Cross-sectional CAD view of motor, coupler, shaft, and bearing configuration. (C) Detailed view of electrode shaft design. (D) Cross-sectional view highlighting flexible motor housing. (E) Full additively manufactured rotation disk electrode overview.

The overall design for the AMRDE system is presented in Figure 2, along with various cross-sectional views, highlighting individual areas of the system. For its design to be successful, the RDE system must meet the following criteria: (1) be constructed using only readily available parts and additively manufactured (3D printed) parts; (2) be significantly less expensive than commercial solutions; (3) use readily available tools for construction; (4) control and maintain the revolutions per minute (RPM) of the electrode; (5) have minimal vibration on the shaft; (6) isolate the system’s electrical noise from the electrode’s output connection; (7) successfully produce electrochemical results comparable to a commercial system; (8) allow the use of commercial electrodes; (9) be simple in operation and build. Additionally, from user experience with commercial RDE systems, several “improvements” were suggested: (10) all-in-one unit (no separate controller with connecting cables); (11) onboard screen; (12) no fasteners on the bottom of the unit to prevent corrosion in open cells.

A modular approach was taken to the design of the RDE system, with it being broken into three main sections: the contacts section contained the end cone, electrical contractors for the electrode and the shaft bearings; the motor section contained the DC motor, rotary encoder, shaft coupler, and motor controller; the top section contained the control electronics, display, outputs, and control knob. This approach allows a user to modify individual components should they not have access to the exact components used in this research; for example, the top lid and knob parts can easily be modified for whatever potentiometer is accessible. Once printed, all 2.5 mm holes were tapped with an M3 tap wrench. The M2.5 screws holding the h-bridge driver board were designed to cut their own thread. The brushed 12 V DC motor with in-built rotary encoder was chosen due to several factors:

- It was readily available from Amazon, which made the project more accessible.
- Low cost of brushed DC motors in comparison to BLDC, 3-phase etc.
- In-built rotary encoder wheel reduces system complexity and cost:

- Specified RPM of 10 000 rpm at 12 VDC matches the specification of the commercial model used for comparison.
- 12 VDC is a common voltage of DC power supply; users are likely to have spare or old 12 VDC power supplies.
- Encoder uses 5 V logic, compatible with low cost MCUs such as Arduino.

The L298N H-bridge motor driver was chosen due to its simplicity, very low cost, and compatibility with Arduino MCUs, though any brushed DC motor controller that supports PWM control would be perfectly suitable, for example, the MAX14870. An Arduino Nano was used as the microcontroller for the system due to its low cost and the abundance of libraries available for the Arduino Development Environment, much simplifying the process of coding devices such as the OLED display. To ensure the motor and controller circuits stay isolated from the electrochemical circuits, a solid ABS coupler was designed to connect the motor shaft to the electrode shaft. This serves to both electrically insulate and adapt the 1.5 mm motor shaft to the 5 mm electrode brass shaft using M3 grub screws to secure both sides. Ideally this coupler would be turned from Delrin or a similar electrical insulator using a lathe to ensure concentricity of the rotating parts.

Commercial electrode rotators use liquid mercury to create an electrical connection between the rotating shaft and the potentiostat connections. However, this poses serious safety concerns due to mercury exposure in a “homemade” RDE setup, and the efficacy of containing mercury in AM containers has not been sufficiently tested/researched to safely implement. Some lower cost RDE setups on the market use silver/carbon brushed contacts, and so low-cost carbon brushes and a brass shaft were used for the RDE design. The carbon brushes chosen were very low-cost replacements for electric drills and power tools, readily available online and from local DIY retailers. These carbon brushes (Figure 2A) are square faced by default, so a concave shape must be ground into the face of the brush that will contact the brass shaft, to ensure the best possible electrical connection when the shaft is rotating. This can be done by preassembling the motor and brush housings and simply running the system until friction wears the correct shape into the face; however, it was found more efficient to manually shape the brushes with emery paper fixed to a brass rod of the same size and then run the motor/shaft to perfect the contact shape. This prevented “chattering” of the contacts and reduced electrical noise in the electrode signal. Once shaped, the rear brass contacts of the brushes were soldered to wires that are passed directly to the banana socket output (the de facto standard connection of electrochemical equipment) of the system through a channel in the printed housing. The shaft was constrained using two bearings (one on either side of the contacts) to reduce vibration in the shaft at the points of electrical contact.

The main shaft of a commercially available RDE was measured and reverse-engineered using digital callipers and thread gauges, and the threaded electrode mating portion was recreated in Fusion360. This section was added to the overall CAD file and was finally turned on a lathe from 5 mm brass rod stock. The technical drawing for the brass shaft can be found in Figure S1. A M4 × 0.7RH thread was cut into the shaft to mate with the commercial electrodes (Figure 2C) available for commercial systems with M4 electrode mounting. This ensured the possibility to use both commercial and bespoke electrodes on the same system. The motor housing is designed to decouple the motor from the rest of the equipment to reduce vibrations and thus was printed using a flexible elastomer in a gyroid infill pattern, which is designed to give an equal distribution of strength (and therefor flexibility) in prints. This housing was screwed into the lower housing, and the L298N motor controller was fixed above it using M2.5 screws, which form their own threads in the printed holes. The upper housing had the Arduino Nano, banana socket, OLED display, and 2.1 mm DC socket installed, and the wiring was completed according to Figure 1A with 24AWG silicone wire, though any wire can reasonably be substituted here. Note that any potentiometer can be used for the speed control, as it is used as a voltage divider between the supply voltage and 0 V. While a higher value potentiometer (or one with a higher number of turns) may give a finer resolution on the speed control, any value is suitable. O-ring stock was cut to length and installed underneath the rpm control knob with CA glue, contacting the top housing, in an effort to reduce accidental movement of the knob through vibration caused by the motor. All parts of the housing are then screwed together with M3 screws; however, the end cone of the RDE is designed to twist onto a printed thread on the bottom of the device. This serves to protect all hardware and fasteners from the chemicals being tested and allows specialist tips to be created for specific experimental setups such as sealing into a three-necked flask. The only exposed component is a stainless-steel bearing, specifically chosen for its shielded construction, preventing ingress into the bearing. The Arduino was then programmed over USB from the Arduino IDE, and system functionality was checked using a laser tachometer to verify the displayed rpm matched the shaft rpm.

Commercial RDE setups can be purchased, costing on average between £4–5000 for the electrode spinner and control box. Commercial working electrodes compatible with these systems are commonly purchased separately, from approximately £300 for carbon to over £500 for platinum or gold disk electrodes. While the ABS parts in this project were printed using a Raise3D E2 printer (purchased from Create Education (Chorley, U.K.) for £2349.00 (+VAT)), the design presented could be produced in PLA or PETG on a sub £200 FFF additive manufacturer (3D printer) from the likes of Creality, Anycubic, Biqu or any budget manufacturer. There are several assumptions made in the process of costing the additive manufacturing rotating disk electrode setup. It is assumed that the researcher has access (whether internal to the research institute or locally) to a lathe or accurate method of shaping the brass round stock used for the RDE shaft. The design requires tapping of 3D-printed holes for fasteners; thus, it is assumed the researcher has access to an M3 tap and tap wrench. The cost of hand tools (allen keys, emery paper, CA glue, scalpel/scissors) is negated, as the tools are common supplies likely already in possession of the researcher.

Table 1 shows the inclusive cost of producing the proposed additive manufactured (3D printed) RDE system (inclusive of one additive manufactured electrode). The additive manufactured (3D printed) RDE system costs less than 2% of the commercial solution (1.72%). With the majority of parts being available from worldwide online retailers, this price should remain fairly constant to all researchers. Note that parts choice is not fixed; in the case of unavailability of parts, informed substitutions can be made readily. For example, should an Arduino Nano not be available, any development board with suitable processing power can be substituted such as an ESP32. This is a lower-cost board that could also enable Wi-Fi or Bluetooth functionality if programmed as such.

**Table 1:** Breakdown of the cost of manufacturing the additive manufacturing rotating disk electrode system^a^

part	cost price/amount including VAT	quantity used	price including VAT
Raise3D ABS filament	£32.39 ($39.21)/kg	169 g	£5.47 ($6.62)
RS Pro Flex 45 filament	£32.69 ($39.57)/500 g	20 g	£0.65 ($0.79)
2 mm O-ring stock	£12.12 ($14.67)/8.5 m	116 mm	£0.17 ($0.21)
Flanged bearings	£8.54 ($10.34)/each	2	£17.08 ($20.68)
Brass 5 mm rod	£32.82 ($39.73)/2.5 m	120 mm	£1.58 ($1.91)
M2.5 × 12 machine screws	£9.40 ($11.38)/50	10	£1.88 ($2.28)
M3 × 3 grub screw	£15.36 ($18.59)/50	4	£1.23 ($1.49)
M3 × 10 machine screw	£20.18 ($24.43)/50	16	£6.46 ($7.82)
10,000 rpm 12 V DC motor	£12.99 ($15.73)	1	£12.99 ($15.73)
Carbon brushes	£5.99 ($7.25)/4	2	£3.00 ($3.63)
L298N H-bridge motor driver	£16.99 ($20.57)/5	1	£3.40 ($4.12)
128 × 64 I2C OLED Display	£14.99 ($18.15)/5	1	£3.00 ($3.63)
Arduino Nano	£17.42 ($21.09)/each	1	£17.42 ($21.09)
Banana socket	£7.15 ($8.66)/5	1	£1.43 ($1.73)
2.1 mm DC barrel socker	£6.48 ($7.84)/each	1	£6.48 ($7.84)
200k trim potentiometer	£2.23 ($2.70)/each	1	£2.23 ($2.70)
		Total cost:	£84.47 ($102.26) (not including assembly time)

a Note that the prices in U.S. dollars are correct as of Aug 17, 2022, and the exchange rate conversion will fluctuate. Prices for items are also likely to vary between countries. Prices included are correct for the United Kingdom as of Aug 17, 2022.

### Design of Additively Manufactured Electrodes

**Figure 3:**
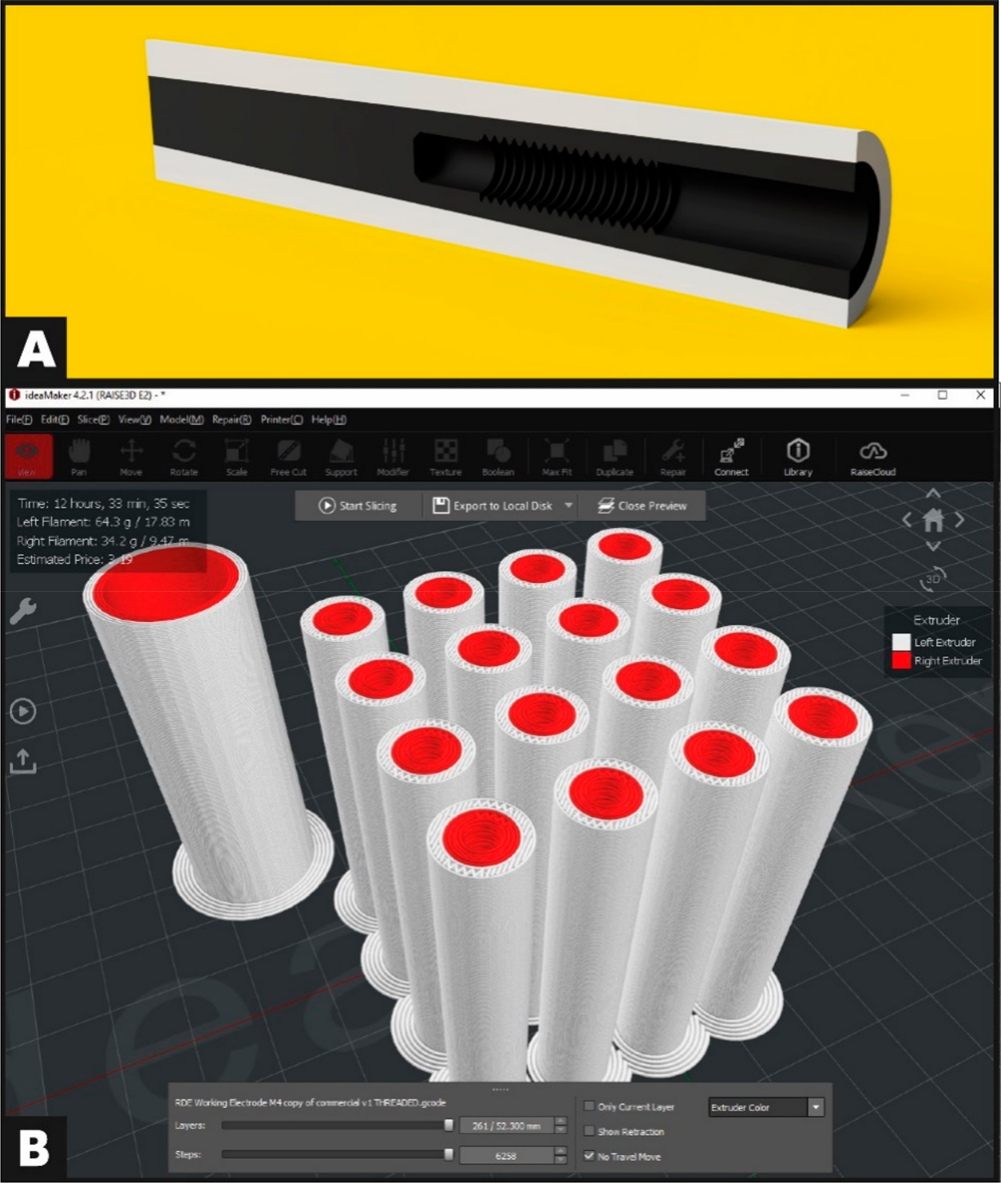
(A) Cross-sectional CAD view of the AM electrodes. (B) Screen capture of the IdeaMaker generated .GCODE file of 16 AM electrodes.

The shaft of a commercial RDE was measured using digital callipers, and the profile of the electrode mating surfaces was recreated in negative in Fusion360. A commercial glassy carbon disk electrode (3 mm diameter) was reverse-engineered, and the overall dimensions were used to create an additively manufactured (3D printed) electrode with the same electrode surface area. However, instead of using a brass insert (electrically connected to the active material) to connect to the shaft, additive manufacturing allows the active material to directly mate with the shaft, by printing an M4 thread into the electrode, Figure 3A. These electrodes were then printed using a Raise3D E2 IDEX printer, with a print profile set to 100% concentric infill, using Raise3D premium PLA for the insulating sleeve and Protopasta conductive (carbon black) PLA for the working electrode. Once printed, an M4 tap was run through the prints to finalize the thread forming; the printing of the threads ensured a much higher level of concentricity than manually tapping an undersized hole to the correct thread, but the stratified manner of construction of FFF prints requires slight refinement of curved surfaces to be accurate. The calculated cost of a single electrode was £0.25 including VAT, from values of 2.9 g (Raise3D PLA) and 1.5 g (CB PLA), in comparison with a commercial glassy carbon disk electrode for prices over £500.00. This places the AME at approximately 0.05% the cost of the GCE.

### Electrochemical Characterization of the Additive Manufacturing Rotating Disk Electrode (AMRDE) vs Commercial Setup

The RDE is a classical hydrodynamic electrochemical technique where control of the mass transport of reactants is achieved through control of the rotational speed. The expression for the limiting current, known as the Levich equation is^1^
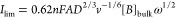
1where *B* is the bulk concentration of the species undergoing electrolysis in the presence of sufficient supporting electrolyte to suppress any mitigation effects (mol cm^–3^), *A* is the electrode area, *n* is the number of electrons transferred per molecule in the half reaction of *B*, *F* is the Faraday constant (C/mol), ω is the rotational speed of the electrode (rad s^–1^), *v* is the kinematic viscosity (cm^2^ s^–1^), and *D* is the diffusion coefficient (cm^2^ s^–1^). From eq 1, we can see that the transport limited current (*I*_lim_) is dependent on the rotation speed (ω^1/2^) of the electrode. This ability makes the RDE setup incredibly useful for electroanalysis, studying reaction rates, corrosion, and fuel cell materials, as extended exposure of the active material can be simulated. Note that the “0.62” is used when the rotating speed is for when ω is in rad s^–1^ but can be changed if ω is measured in rpm (0.20) or in Hz (1.554).

**Figure 4:**
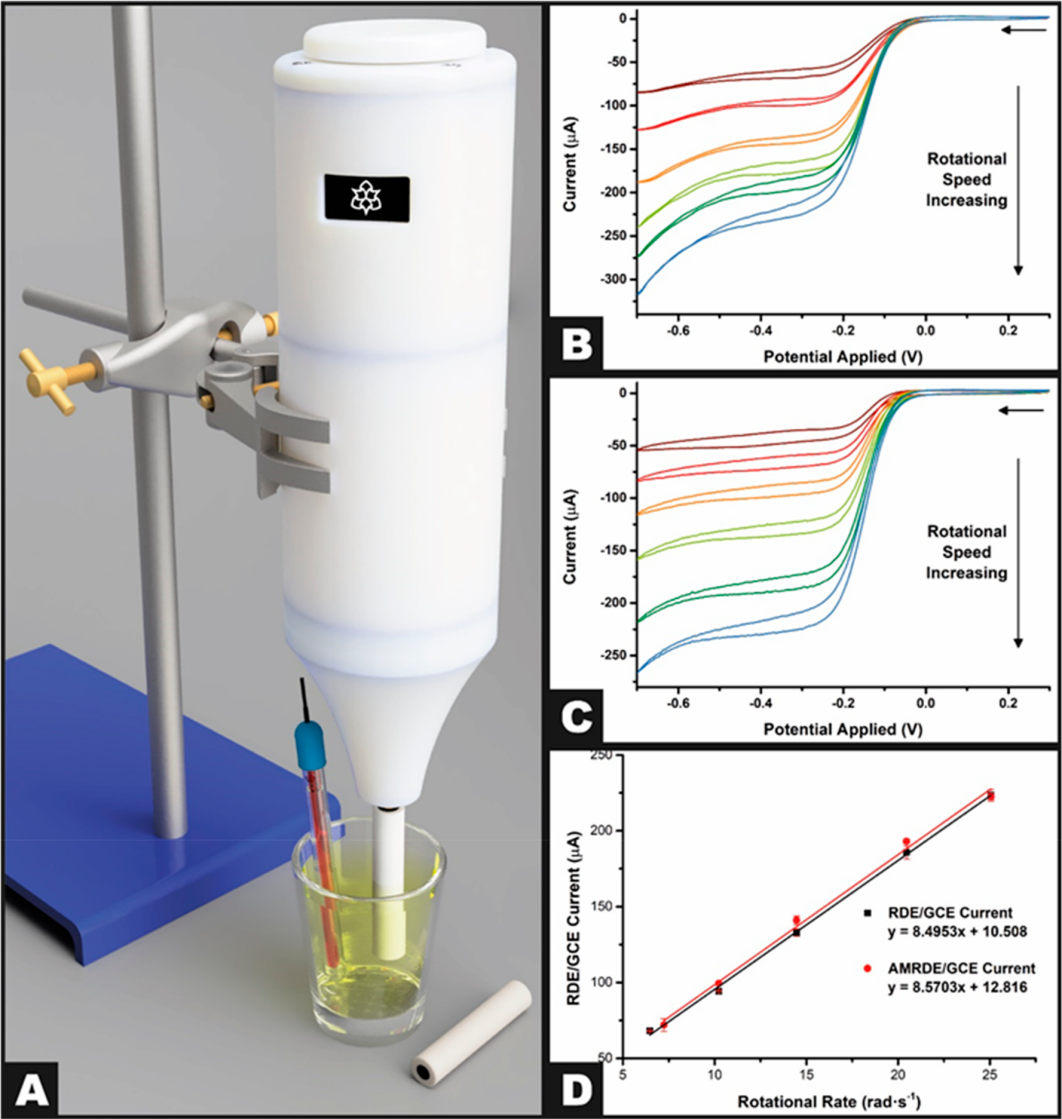
(A) Schematic of the experimental setup used in the experiments. (B) Cyclic voltammograms of hexaamineruthenium(III) chloride (1 mM) in KCl (0.1 M) using the additively manufactured rotation disk electrode setup with a commercial GCE, nichrome wire coil counter electrode, and Ag|AgCl reference electrode. (C) Cyclic voltammograms of hexaamineruthenium(III) chloride (1 mM) in KCl (0.1 M) using a commercial rotating disk electrode setup with a commercial GCE, nichrome wire coil counter electrode, and Ag|AgCl reference electrode. (D) Levich plots for the additively manufactured and commercial RDE rotating disk electrode setups.

Shown in Figure 4A is a schematic of the AMRDE. We turn to benchmark of the AMRDE which has been equipped with a commercially glassy carbon electrode using the near-ideal outer-sphere redox probe hexaamineruthenium(III) chloride (RuHex, 1 mM in 0.1 M KCl) which is shown in Figure 4B at various rotation rates (52.3–628.3 rad s^–1^) and for comparison the response of a full commercially RDE equipment with a glassy carbon electrode (Figure 4C). It can be seen that for both systems a limiting current is reached of increasing magnitude with increased rotation rates. These values can be utilized to produce the corresponding Levich plots for each system, as seen in Figure 4D, where both systems show linearity and good agreement, providing evidence toward the effectiveness of the AMRDE experimental system. The gradient of this plot can be used to calculate the diffusion coefficient of the system under analysis, with the AMRDE and commercial experimental systems exhibiting 8.03 (±0.03) × 10^–6^ and 8.11 (±0.51) × 10^–6^ cm^2^ s^–1^, respectively, showing good agreement with the literature.^20^ These data show that an AMRDE system can produce comparable results to a commercially purchased one, at less than 2% of the cost.

**Figure 5:**
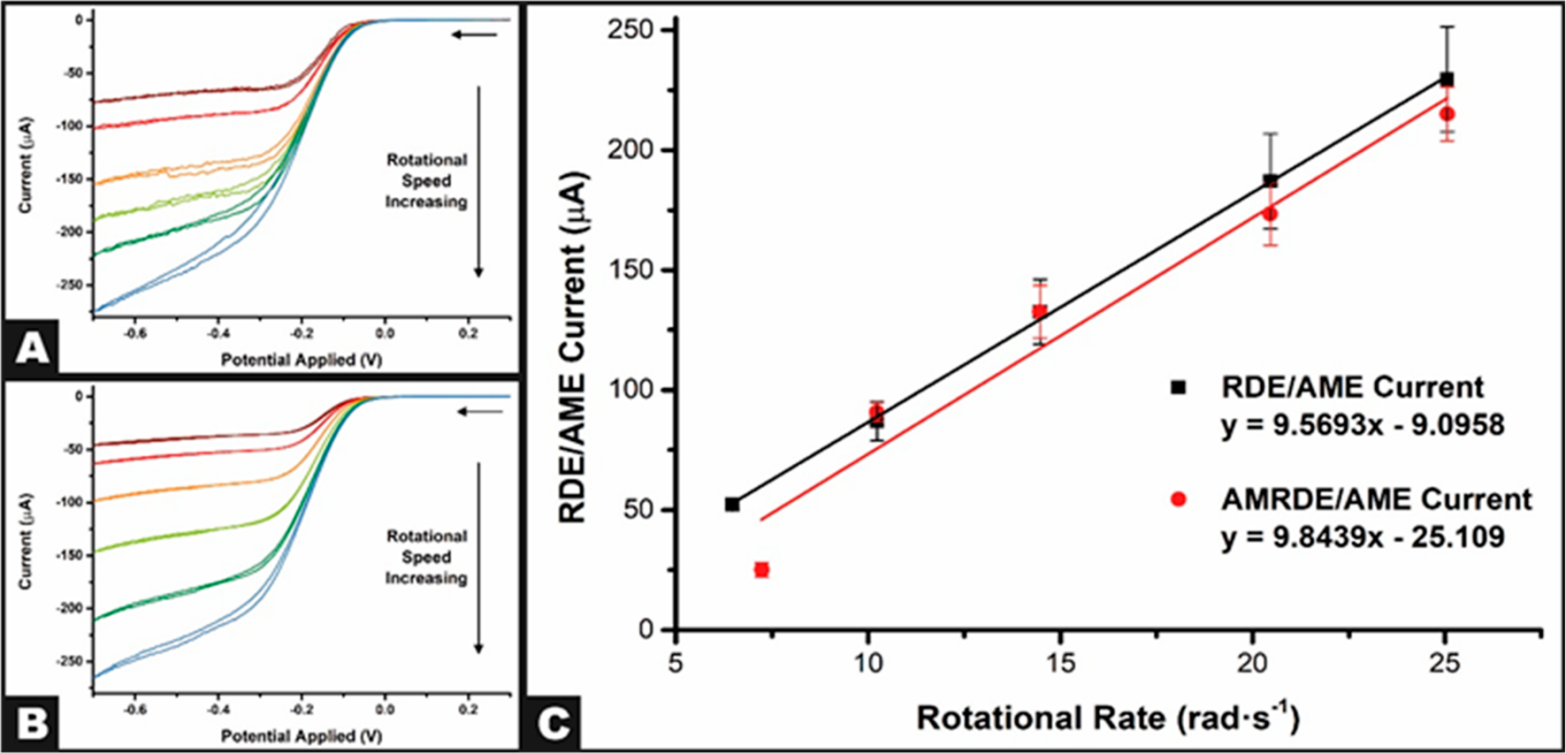
(A) Cyclic voltammograms of hexaamineruthenium(III) chloride (1 mM) in KCl (0.1 M) using the additive manufacturing rotating disk electrode setup with an additive manufacturing electrode, nichrome wire coil counter electrode, and Ag|AgCl reference electrode. (B) Cyclic voltammograms of hexaamineruthenium(III) chloride (1 mM) in KCl (0.1 M) using a commercial rotating disk electrode setup with an additive manufacturing electrode, nichrome wire coil counter electrode, and Ag|AgCl reference electrode. (C) Levich plots for the additively manufactured and commercial rotating disk electrode setups.

Next, we consider the response of the AMRDEw that has been equipped with an AME, and then compared with the glassy carbon electrode towards the redox probe hexaamineruthenium(III) chloride (RuHex, 1 mM in 0.1 M KCl). The cyclic voltammetry responses obtained at an AME rotating at various speeds (52.3–628.3 rad s^–1^) using an AM and commercial experimental system are presented in Figure 5A and Figure 5B, respectively. Once again, the AM experimental system shows excellent agreement with the commercially purchased option, with the limiting currents measured increasing linearly with the rotational rate. When plotted into the appropriate Levich plot, these graphs show excellent correlation with those obtained for the GCE, highlighting how the production and use of AMEs in RDE experiments can be a viable option, especially at 0.05% of the cost. Using the gradients of the Levich plots, it was found that by using an AME on both the AM and commercial experimental systems, diffusion coefficients of *D* = 9.31 (±0.46) × 10^–6^ and 9.05 (±0.23) × 10^–6^ cm^2^ s^–1^ were obtained, again showing excellent agreement with the literature.^21^ This provides further evidence supporting the use of the AM experimental system but also encourages the use of AMEs in RDE experiments due to the simplicity of production, possibilities in terms of electrode design, and ultralow cost per electrode. These mainly AM complete systems could provide lower-income groups across the globe the accessibility to perform reliable RDE measurements. To further emphasize the performance of the AM system, we turn to a possible application of electroanalytical sensing.

### Application to the Detection of L-DOPA

**Figure 6:**
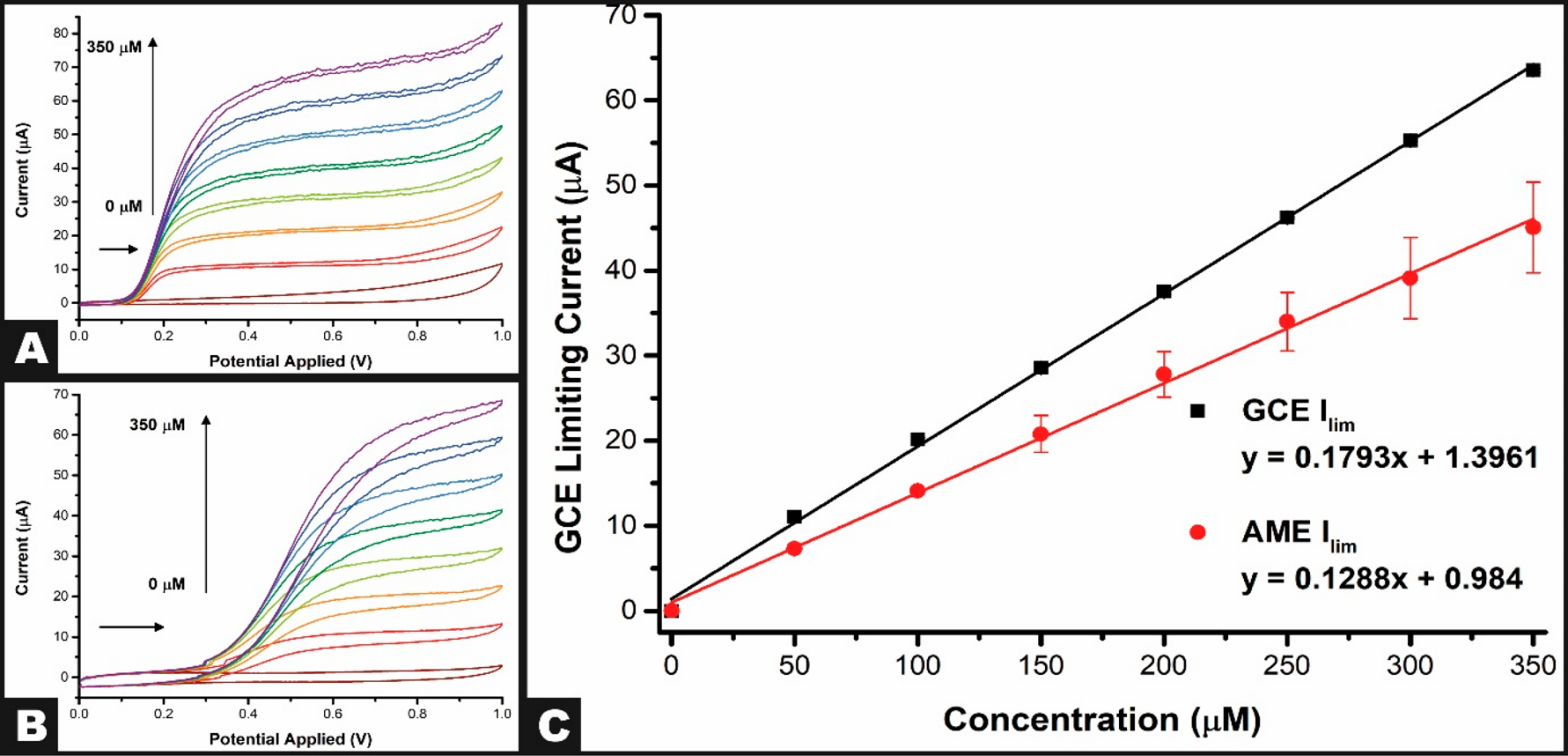
(A) Cyclic voltammograms of the addition of levdopa (0–350 μM) in PBS (0.01 M) using the additively manufactured rotating disk electrode setup with an additive manufacturing electrode at 418.9 rad s^–1^, nichrome wire coil counter electrode, and Ag|AgCl reference electrode. (B) Cyclic voltammograms of the addition of L-DOPA (0–350 μM) in PBS (0.01 M) using the additively manufactured RDE setup with a commercial GCE at 418.9 rad s^–1^, nichrome wire coil counter electrode, and Ag|AgCl reference electrode. (C) Plots of the additively manufactured rotating disk electrode limiting current versus the concentration of L-DOPA at 418.9 rad s^–1^.

Levodopa (L-DOPA) is a drug used to control bradykinetic symptoms in Parkinson’s disease, a progressive and incurable neurological disorder. Due to the short half-life of the drug, patients require complex drug regimens that need monitoring.^22^ It has been shown that the oxidation of L-DOPA can achieve greater peak currents at more acidic pH;^23^ however, due to the advantages of RDE systems, we will show the detection at neutral pH 7.4. Figure 6A exhibits, for the first time, the detection of L-DOPA at the AMRDE system. Upon the addition of 50 μM L-DOPA, a clear increase in the measured current is seen to 7.3 (±0.01) μA from the baseline. This increase continues until a current value of 45 (±5) μA is reached for the addition of 350 μM. This showed good agreement with results when using a commercial GCE, Figure 6B, where for 50 μM, a current of 11.0 (±0.2) μA was obtained, increasing to 63.5 (±0.2) μA for the addition of 350 μM. In both cases, a linear calibration plot was obtained as presented in Figure 6C, where the GCE is shown to provide improved sensitivity in comparison to the AME; note that the different materials will exhibit reaction kinetics. Utilizing these plots and the 3σ method, limits of detection for both systems were calculated to be 0.23 (±0.05) μM and 0.17 (±0.01) μM for the AME and GCE, respectively. Note that the comparison between AMRDE and Commercial RDE is made using both commercial glassy carbon electrodes and AM electrodes. Both systems yielded similar sensitivity, thus validating the AMRDE system (Supporting Information). Additionally, experiments utilizing the cathodic peak were performed for the detection of phosphate, Figure S3. This work highlights how AM can be utilized to produce high performing functional electrochemical equipment at significantly reduced costs, making this field of work increasingly accessible to research groups across the world.

## Conclusions

This work describes the design, production, and characterization of an additively manufactured rotating disk electrode experimental system for electrochemical and electroanalytical applications. Additionally, additive manufactured electrodes are produced and characterized against a commercially purchased glassy carbon electrode (GCE). The design constraints, manufacturing, and coding of the additive manufactured experimental system are discussed, showing that the production of a high performing system is capable for under £84.47 ($102.26), which could be decreased further depending on available suppliers. The performance of this system was benchmarked against a commercially purchased RDE system with a GCE, with both systems producing excellent agreement between diffusion coefficients for hexaamineruthenium(III) chloride. Furthermore, it was shown that utilizing an additive manufactured electrodes against this near-ideal outer-sphere redox probe garnered agreement with each other and literature values. The AM systems, both experimental and electrode, were then utilized toward the electroanalytical detection of the Parkinson’s disease drug, levodopa (L-DOPA). When used in conjunction with each other, it was found that L-DOPA could be determined with a limit of detection of 0.23 (±0.05) μM. This work highlights the benefits that additive manufacturing can have for an electrochemical research laboratory, allowing the bespoke production of high-quality equipment. This work shows how AM can make this area of research more accessible for researchers all around the world.

## Supporting Information Available

Details of the measurement of L-DOPA and the rotating disk electrode code (PDF)
Video of how the rotating disk electrode system was constructed (AVI)

## Supplementary Material

ac2c02884_si_001.pdf

ac2c02884_si_002.avi
