# Supplementary material for: Additively Manufactured Rotating Disk Electrodes and Experimental Setup
Source: Anal Chem. 2022 Sep 21;94(39):13540–8. doi: 10.1021/acs.analchem.2c02884 (PMC9535625; doi:10.1021/acs.analchem.2c02884)
Supplement: Supplementary file 1 — ac2c02884_si_001.pdf [file ac2c02884_si_001.pdf]

Supporting Information for:

Additively Manufactured Rotating Disk Electrodes and Experimental Setup

Matthew J. Whittingham, Robert D. Crapnell and Craig E. Banks*

Faculty of Science and Engineering, Manchester Metropolitan University, Chester Street, M1 5GD, United Kingdom.

*To whom correspondence should be addressed.
E-mail: c.banks@mmu.ac.uk; Tel: +44(0)1612471196

All print files for the production of this set-up can be found through the following link: https://www.printables.com/model/255406-3d-printed-rotating-disk-electrode-setup

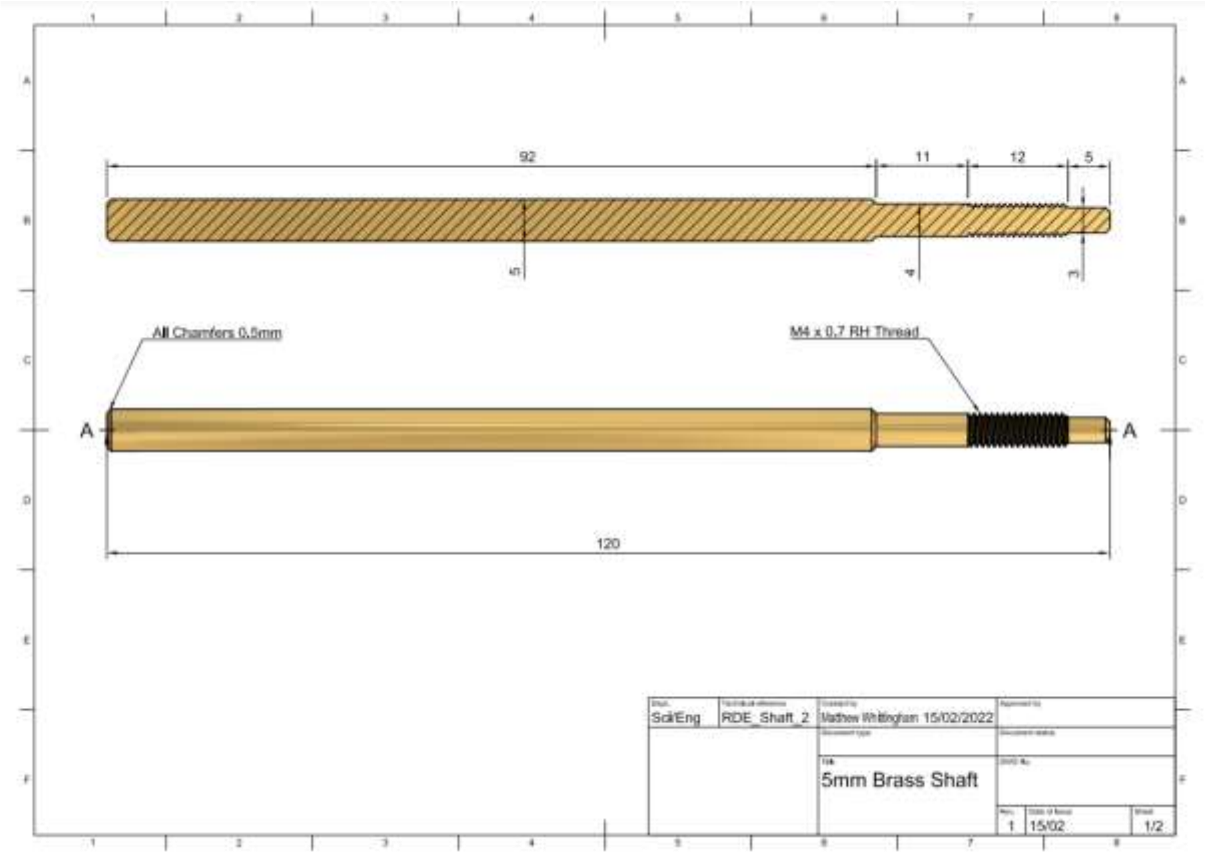

Figure S1. Technical drawing for the production of the brass shaft for the Additive Manufacturing Rotating Disk Electrode Set-up.

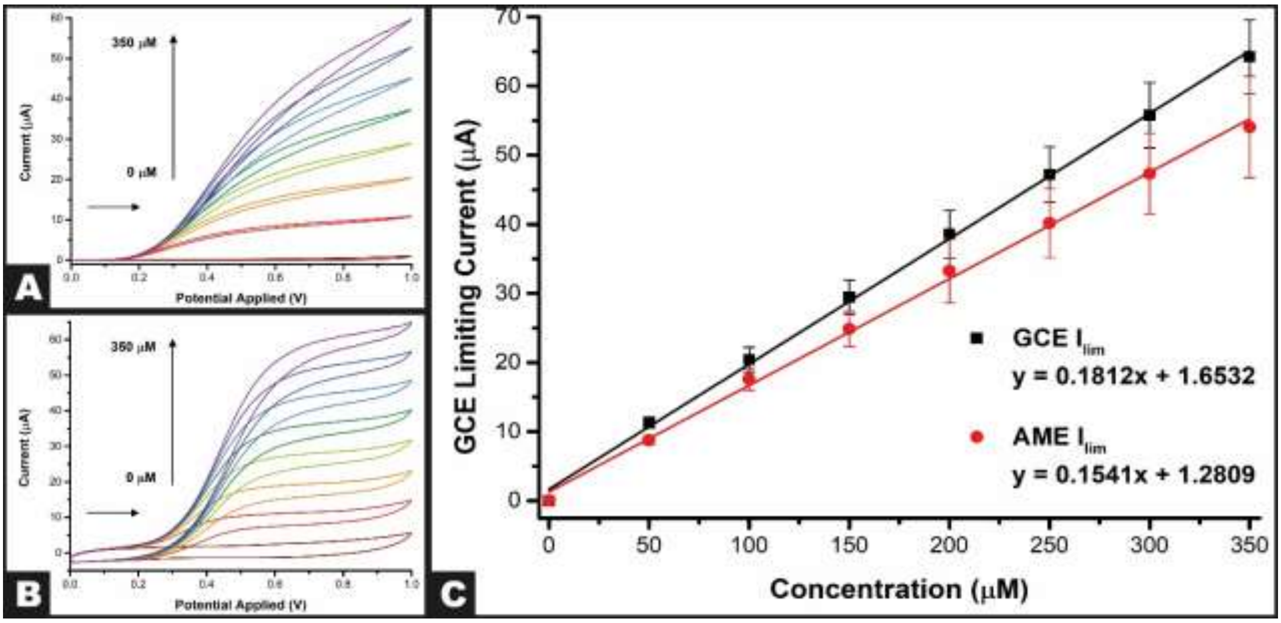

Figure S2. A) Cyclic voltammograms of the addition of Levdopa (0-350 µM) in PBS (0.01 M) using a commercial RDE setup with an AME at 418.9 rad s^{-1}, nichrome wire coil counter electrode and Ag|AgCl reference electrode. **B)** Cyclic voltammograms of the addition of L-DOPA (0-350 µM) in PBS (0.01 M) using a commercial RDE setup with a commercial GCE at 418.9 rad s^{-1}, nichrome wire coil counter electrode and Ag|AgCl reference electrode. **C)** Plots of the Commercial RDE limiting current versus the concentration of L-DOPA at 418.9 rad s^{-1}.

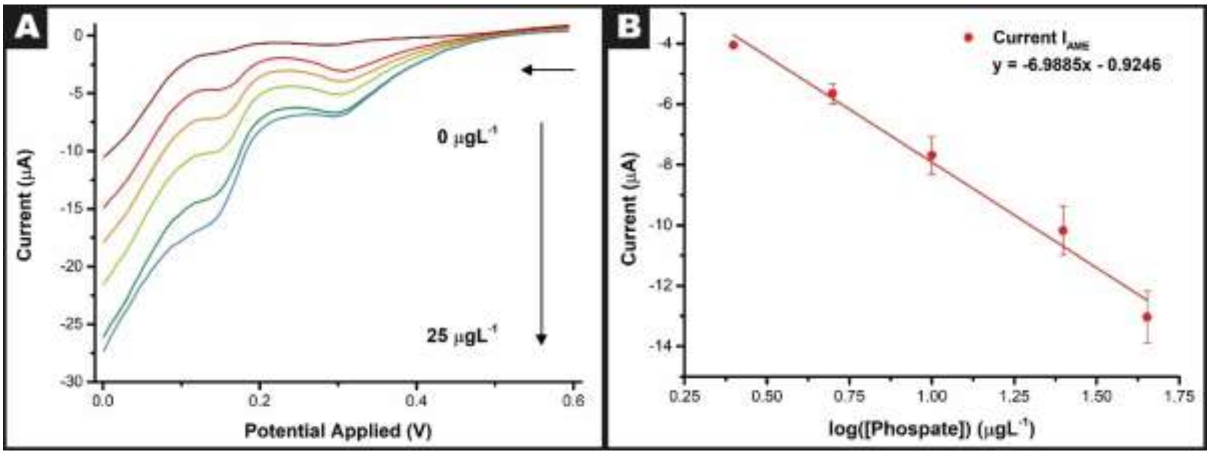

Figure S3. A) Linear sweep voltammograms of the addition of phosphate (1 – 25 µg L^{-1}) to ammonium molybdate tetrahydrate (640 mg L^{-1}) using the Additive Manufacturing Rotating Disk set-up with an AME at 209.44 rad s^{-1}, nichrome wire coil counter electrode and Ag|AgCl reference electrode. **B)** Plot of the LSV current versus the log of the concentration of phosphate at 209.44 rad s^{-1}.

RDE Code

```
//Additively Manufactured Rotating Disk Electrode Experimental Setup

//Electrode Spinner Firmware

//Matthew J. Whittingham

//Manchester Metropolitan University


//include libraries

#include <Arduino.h>

#include <avr/interrupt.h>

#include <stdio.h>

#include <Wire.h>

#include <Adafruit_GFX.h>

#include <Adafruit_SSD1306.h>


#define SCREEN_WIDTH 128 // OLED display width, in pixels

#define SCREEN_HEIGHT 64 // OLED display height, in pixels


inline void DisplayStuff(uint16_t rpm);


Adafruit_SSD1306 display(SCREEN_WIDTH, SCREEN_HEIGHT, &Wire, -1); //define screen specs.


//define MMU logo bitmap for bootscreen

static const unsigned char PROGMEM image_data_mmulogo[] = {

  0x00, 0x00, 0x00, 0x00, 0x00, 0x00, 0x00, 0x00, 0x00, 0x00, 0x00, 0x00, 0x00, 0x00, 0x00, 0x00,

  0x00, 0x00, 0x00, 0x00, 0x00, 0x00, 0x00, 0x01, 0x80, 0x00, 0x00, 0x00, 0x00, 0x00, 0x00, 0x00,

  0x00, 0x00, 0x00, 0x00, 0x00, 0x00, 0x00, 0x07, 0xe0, 0x00, 0x00, 0x00, 0x00, 0x00, 0x00, 0x00,

  0x00, 0x00, 0x00, 0x00, 0x00, 0x00, 0x00, 0x0f, 0xf0, 0x00, 0x00, 0x00, 0x00, 0x00, 0x00, 0x00,

  0x00, 0x00, 0x00, 0x00, 0x00, 0x00, 0x00, 0x3f, 0xf8, 0x00, 0x00, 0x00, 0x00, 0x00, 0x00, 0x00,

  0x00, 0x00, 0x00, 0x00, 0x00, 0x00, 0x00, 0x3e, 0x7c, 0x00, 0x00, 0x00, 0x00, 0x00, 0x00, 0x00,

  0x00, 0x00, 0x00, 0x00, 0x00, 0x00, 0x00, 0xfc, 0x3e, 0x00, 0x00, 0x00, 0x00, 0x00, 0x00, 0x00,

  0x00, 0x00, 0x00, 0x00, 0x00, 0x00, 0x00, 0xf8, 0x1f, 0x00, 0x00, 0x00, 0x00, 0x00, 0x00, 0x00,

  0x00, 0x00, 0x00, 0x00, 0x00, 0x00, 0x01, 0xf0, 0x0f, 0x80, 0x00, 0x00, 0x00, 0x00, 0x00, 0x00,

  0x00, 0x00, 0x00, 0x00, 0x00, 0x00, 0x03, 0xe0, 0x07, 0xc0, 0x00, 0x00, 0x00, 0x00, 0x00, 0x00,

  0x00, 0x00, 0x00, 0x00, 0x00, 0x00, 0x03, 0xc0, 0x03, 0xe0, 0x00, 0x00, 0x00, 0x00, 0x00, 0x00,
```

```
0x00, 0x00, 0x00, 0x00, 0x00, 0x00, 0x07, 0x80, 0x01, 0xe0, 0x00, 0x00, 0x00, 0x00, 0x00, 0x00,
0x00, 0x00, 0x00, 0x00, 0x00, 0x00, 0x07, 0x00, 0x00, 0xe0, 0x00, 0x00, 0x00, 0x00, 0x00, 0x00,
0x00, 0x00, 0x00, 0x00, 0x00, 0x00, 0x0f, 0x00, 0x00, 0xf0, 0x00, 0x00, 0x00, 0x00, 0x00, 0x00,
0x00, 0x00, 0x00, 0x00, 0x00, 0x00, 0x0f, 0x80, 0x01, 0xf0, 0x00, 0x00, 0x00, 0x00, 0x00, 0x00,
0x00, 0x00, 0x00, 0x00, 0x00, 0x00, 0x0f, 0xe0, 0x07, 0xf0, 0x00, 0x00, 0x00, 0x00, 0x00, 0x00,
0x00, 0x00, 0x00, 0x00, 0x00, 0x00, 0x0f, 0xf8, 0x1f, 0xf0, 0x00, 0x00, 0x00, 0x00, 0x00, 0x00,
0x00, 0x00, 0x00, 0x00, 0x01, 0x00, 0x07, 0xf8, 0x1f, 0xe0, 0x00, 0x80, 0x00, 0x00, 0x00, 0x00,
0x00, 0x00, 0x00, 0x00, 0x01, 0xff, 0xff, 0xfc, 0x3f, 0xff, 0xff, 0x80, 0x00, 0x00, 0x00, 0x00,
0x00, 0x00, 0x00, 0x00, 0x00, 0xff, 0xff, 0x9e, 0x79, 0xff, 0xff, 0x00, 0x00, 0x00, 0x00, 0x00,
0x00, 0x00, 0x00, 0x00, 0x00, 0x7f, 0xff, 0x1e, 0x78, 0xff, 0xfe, 0x00, 0x00, 0x00, 0x00, 0x00,
0x00, 0x00, 0x00, 0x00, 0x00, 0x7f, 0xfe, 0x0f, 0xf0, 0x7f, 0xfe, 0x00, 0x00, 0x00, 0x00, 0x00,
0x00, 0x00, 0x00, 0x00, 0x00, 0x3e, 0x00, 0x0f, 0xe0, 0x00, 0x3c, 0x00, 0x00, 0x00, 0x00, 0x00,
0x00, 0x00, 0x00, 0x00, 0x00, 0x1f, 0x00, 0x07, 0xe0, 0x00, 0x78, 0x00, 0x00, 0x00, 0x00, 0x00,
0x00, 0x00, 0x00, 0x00, 0x00, 0x0f, 0x80, 0x07, 0xe0, 0x00, 0xf8, 0x00, 0x00, 0x00, 0x00, 0x00,
0x00, 0x00, 0x00, 0x00, 0x00, 0x0f, 0xc0, 0x03, 0xc0, 0x01, 0xf0, 0x00, 0x00, 0x00, 0x00, 0x00,
0x00, 0x00, 0x00, 0x00, 0x00, 0x07, 0xc0, 0x03, 0xc0, 0x03, 0xe0, 0x00, 0x00, 0x00, 0x00, 0x00,
0x00, 0x00, 0x00, 0x00, 0x00, 0x03, 0xc0, 0x03, 0xc0, 0x03, 0xc0, 0x00, 0x00, 0x00, 0x00, 0x00,
0x00, 0x00, 0x00, 0x00, 0x00, 0x03, 0xc0, 0x03, 0xc0, 0x03, 0xe0, 0x00, 0x00, 0x00, 0x00, 0x00,
0x00, 0x00, 0x00, 0x00, 0x00, 0x07, 0xc0, 0x03, 0xc0, 0x03, 0xe0, 0x00, 0x00, 0x00, 0x00, 0x00,
0x00, 0x00, 0x00, 0x00, 0x00, 0x1f, 0xc0, 0x03, 0xc0, 0x03, 0xf8, 0x00, 0x00, 0x00, 0x00, 0x00,
0x00, 0x00, 0x00, 0x00, 0x00, 0x3f, 0xc0, 0x07, 0xe0, 0x03, 0xfc, 0x00, 0x00, 0x00, 0x00, 0x00,
0x00, 0x00, 0x00, 0x00, 0x00, 0xff, 0xc0, 0x1f, 0xf8, 0x03, 0xfe, 0x00, 0x00, 0x00, 0x00, 0x00,
0x00, 0x00, 0x00, 0x00, 0x00, 0xff, 0xc0, 0xff, 0xff, 0x03, 0xff, 0x00, 0x00, 0x00, 0x00, 0x00,
0x00, 0x00, 0x00, 0x00, 0x00, 0xf7, 0xff, 0xfe, 0x7f, 0xff, 0xef, 0x00, 0x00, 0x00, 0x00, 0x00,
0x00, 0x00, 0x00, 0x00, 0x01, 0xe3, 0xff, 0xfc, 0x3f, 0xff, 0xc7, 0x80, 0x00, 0x00, 0x00, 0x00,
0x00, 0x00, 0x00, 0x00, 0x03, 0xe0, 0x7f, 0xf0, 0x0f, 0xfe, 0x07, 0x80, 0x00, 0x00, 0x00, 0x00,
0x00, 0x00, 0x00, 0x00, 0x03, 0xc0, 0x01, 0xe0, 0x07, 0x80, 0x03, 0x80, 0x00, 0x00, 0x00, 0x00,
0x00, 0x00, 0x00, 0x00, 0x03, 0xc0, 0x01, 0xe0, 0x07, 0xc0, 0x03, 0x80, 0x00, 0x00, 0x00, 0x00,
0x00, 0x00, 0x00, 0x00, 0x03, 0xc0, 0x03, 0xc0, 0x03, 0xc0, 0x03, 0xc0, 0x00, 0x00, 0x00, 0x00,
0x00, 0x00, 0x00, 0x00, 0x03, 0x80, 0x07, 0x80, 0x01, 0xe0, 0x01, 0xc0, 0x00, 0x00, 0x00, 0x00,
0x00, 0x00, 0x00, 0x00, 0x03, 0x80, 0x07, 0x00, 0x00, 0xe0, 0x01, 0xc0, 0x00, 0x00, 0x00, 0x00,
0x00, 0x00, 0x00, 0x00, 0x07, 0x80, 0x07, 0x00, 0x00, 0xf0, 0x01, 0xe0, 0x00, 0x00, 0x00, 0x00,
0x00, 0x00, 0x00, 0x00, 0x07, 0x80, 0x0f, 0x00, 0x00, 0xf0, 0x01, 0xe0, 0x00, 0x00, 0x00, 0x00,
0x00, 0x00, 0x00, 0x00, 0x07, 0x80, 0x0f, 0xe0, 0x07, 0xf0, 0x03, 0xe0, 0x00, 0x00, 0x00, 0x00,
```

```
  0x00, 0x00, 0x00, 0x00, 0x03, 0xc0, 0x0f, 0xf0, 0x0f, 0xf0, 0x03, 0xc0, 0x00, 0x00, 0x00, 0x00,
  0x00, 0x00, 0x00, 0x00, 0x03, 0xf0, 0x0f, 0xf8, 0x1f, 0xf0, 0x0f, 0xc0, 0x00, 0x00, 0x00, 0x00,
  0x00, 0x00, 0x00, 0x00, 0x03, 0xfc, 0x07, 0xfc, 0x3f, 0xe0, 0x3f, 0xc0, 0x00, 0x00, 0x00, 0x00,
  0x00, 0x00, 0x00, 0x00, 0x00, 0xff, 0xff, 0xfc, 0x3f, 0xff, 0xff, 0x80, 0x00, 0x00, 0x00, 0x00,
  0x00, 0x00, 0x00, 0x00, 0x00, 0x3f, 0xff, 0xbe, 0x7d, 0xff, 0xfc, 0x00, 0x00, 0x00, 0x00, 0x00,
  0x00, 0x00, 0x00, 0x00, 0x00, 0x0f, 0xff, 0x3e, 0x78, 0xff, 0xf0, 0x00, 0x00, 0x00, 0x00, 0x00,
  0x00, 0x00, 0x00, 0x00, 0x00, 0x00, 0x00, 0x1f, 0x78, 0x00, 0x00, 0x00, 0x00, 0x00, 0x00, 0x00,
  0x00, 0x00, 0x00, 0x00, 0x00, 0x00, 0x00, 0x0f, 0xf0, 0x00, 0x00, 0x00, 0x00, 0x00, 0x00, 0x00,
  0x00, 0x00, 0x00, 0x00, 0x00, 0x00, 0x00, 0x07, 0xe0, 0x00, 0x00, 0x00, 0x00, 0x00, 0x00, 0x00,
  0x00, 0x00, 0x00, 0x00, 0x00, 0x00, 0x00, 0x07, 0xe0, 0x00, 0x00, 0x00, 0x00, 0x00, 0x00, 0x00,
  0x00, 0x00, 0x00, 0x00, 0x00, 0x00, 0x00, 0x07, 0xe0, 0x00, 0x00, 0x00, 0x00, 0x00, 0x00, 0x00,
  0x00, 0x00, 0x00, 0x00, 0x00, 0x00, 0x00, 0x03, 0xc0, 0x00, 0x00, 0x00, 0x00, 0x00, 0x00, 0x00,
  0x00, 0x00, 0x00, 0x00, 0x00, 0x00, 0x00, 0x03, 0xc0, 0x00, 0x00, 0x00, 0x00, 0x00, 0x00, 0x00,
  0x00, 0x00, 0x00, 0x00, 0x00, 0x00, 0x00, 0x03, 0xc0, 0x00, 0x00, 0x00, 0x00, 0x00, 0x00, 0x00,
  0x00, 0x00, 0x00, 0x00, 0x00, 0x00, 0x00, 0x03, 0xc0, 0x00, 0x00, 0x00, 0x00, 0x00, 0x00, 0x00,
  0x00, 0x00, 0x00, 0x00, 0x00, 0x00, 0x00, 0x01, 0x80, 0x00, 0x00, 0x00, 0x00, 0x00, 0x00, 0x00,
  0x00, 0x00, 0x00, 0x00, 0x00, 0x00, 0x00, 0x01, 0x80, 0x00, 0x00, 0x00, 0x00, 0x00, 0x00, 0x00,
  0x00, 0x00, 0x00, 0x00, 0x00, 0x00, 0x00, 0x00, 0x00, 0x00, 0x00, 0x00, 0x00, 0x00, 0x00, 0x00,
  0x00, 0x00, 0x00, 0x00, 0x00, 0x00, 0x00, 0x00, 0x00, 0x00, 0x00, 0x00, 0x00, 0x00, 0x00, 0x00
};

//define integers and variables
volatile int z = 0;
int zstore = 0;
unsigned long time0 = 0;
unsigned long timez = 0;
unsigned long zdiff = 0;
unsigned long time1 = 0;
unsigned long mpr = 0;
int rpm = 0;
int rpmfeedback = 0;
long stop = 0;
int potValue = 0;
int pwmout = 0;
```

```
//volatile register byte upcount asm("r3");  // dedicated a register to ISR var

ISR(INT0_vect) {
  z++;
}

ISR (INT1_vect) {
  z++;
}

// Configure digital pins 9 and 10 as 16-bit PWM outputs.
void setupPWM16(int val) {
    DDRB |= _BV(PB1) | _BV(PB2);        /* set pins as outputs */
    TCCR1A = _BV(COM1A1) | _BV(COM1B1)  /* non-inverting PWM */
        | _BV(WGM11);                   /* mode 14: fast PWM, TOP=ICR1 */
    TCCR1B = _BV(WGM13) | _BV(WGM12)
        | _BV(CS10);                    /* no prescaling */
    ICR1 = val;                         /* TOP counter value */
}

void setup() {

setupPWM16(800);              //ceiling to 800. this gives 16MHz / 800 = 20kHz pwm frequency

pinMode(2, INPUT_PULLUP);      //set input pinmode
pinMode(3, INPUT_PULLUP);      //set input pinmode
pinMode(A0, INPUT);            //set input pinmode

    // activate external interrupts 0&1
  EICRA &= ~(bit(ISC00) | bit (ISC01) | bit (ISC10) | bit (ISC11));     // clear existing flags
  EICRA |= (bit (ISC01) | bit (ISC11));                               // set wanted flags (any change interrupts) ISCX0 is
change, X1 is fall
  EIFR   = (bit (INTF0) | bit (INTF1));                         // clear flag for interrupts
  EIMSK |= (bit (INT0) | bit (INT1));                           // enable them
```

```
Serial.begin(115200);                    //begin serial connection 115200 baudrate for debugging

if(!display.begin(SSD1306_SWITCHCAPVCC, 0x3C)) {        //if screen fails to intitialise
  Serial.println("SSD1306 Allocation Failed");          //tell me screen failed on serial monitor
  for(;;);                                 //don't proceed, loop forever
}

display.clearDisplay();                    //clear display buffer
display.drawBitmap(0, 0, image_data_mmulogo, 128, 64, 1);   //draw boot screen
display.display();                         //display boot screen

//delay(2000);
}

void loop() {

cli();              //uninterruptable loop determines rpm
zstore += z ;
z = 0 ;
sei();
time0 = micros();

if (zstore >= 20) {
  zdiff = time0 - timez;
  mpr = zdiff / zstore;
  rpm = 2726400 / mpr;
  zstore = 0;
  timez = time0;
}

if (timez >= (time1 + 500000) ) {

  //Serial.println(mph);
```

```
  DisplayStuff(rpm);       //print rpm value to screen
  stop = 0;
  time1 += 500000;
} else {
  stop++;
}

if (stop >= 250000) {
  DisplayStuff(0);
  stop = 0;
}

potValue = analogRead(A0);                 //read proportional voltage from pot.
pwmout = map(potValue,0,1023,200,800);      //map read value to PWM value

  noInterrupts();
  OCR1A = 0;                            //analogwrite low to pin 9, only needed for direction control
  OCR1B = pwmout;                          //analogwrite mapped PWM value to pin 10
  interrupts();                          //end uninterruptable phase

}

inline void DisplayStuff(uint16_t rpm)
{
  display.clearDisplay();          //clear display buffer

  display.setTextSize(1);          //text size small
  display.setTextColor(WHITE);      //white text - might not be needed

  display.setCursor(35,10);         //from 35X 0Y
  display.println("3D-PRINTED");     //print text
  display.setCursor(35,20);         //from 35X 10Y
  display.println("RDE SYSTEM");     //print text
```

```
  display.setTextSize(2);          //text size medium
  display.setCursor(30,40);        //from 15X 30Y
  display.print(rpm);              //print rpm value

  display.setTextSize(1);          //text size small
  display.println(" RPM");         //print text after previous, same line

  display.display();               //display buffer
}
```
